# The Earliest Giant *Osprioneides* Borings from the Sandbian (Late Ordovician) of Estonia

**DOI:** 10.1371/journal.pone.0099455

**Published:** 2014-06-05

**Authors:** Olev Vinn, Mark A. Wilson, Mari-Ann Mõtus

**Affiliations:** 1 Department of Geology, University of Tartu, Tartu, Estonia; 2 Department of Geology, The College of Wooster, Wooster, Ohio, United States of America; 3 Institute of Geology, Tallinn University of Technology, Tallinn, Estonia; Raymond M. Alf Museum of Paleontology, United States of America

## Abstract

The earliest *Osprioneides kampto* borings were found in bryozoan colonies of Sandbian age from northern Estonia (Baltica). The Ordovician was a time of great increase in the quantities of hard substrate removed by single trace makers. Increased predation pressure was most likely the driving force behind the infaunalization of larger invertebrates such as the *Osprioneides* trace makers in the Ordovician. It is possible that the *Osprioneides* borer originated in Baltica or in other paleocontinents outside of North America.

## Introduction

The oldest macroborings in the world are the small simple holes of *Trypanites* reported in Early Cambrian archaeocyathid reefs in Labrador [1], [2]. The next oldest macroborings are found in carbonate hardgrounds of Early Ordovician age [3], [4], [5], [6]. There was a great increase in bioerosion intensity and diversity in the Ordovician, now termed the Ordovician Bioerosion Revolution [7]. In the Middle and Late Ordovician, shells and hardgrounds are often thoroughly riddled with holes, most of them attributable to *Trypanites* and *Palaeosabella* [8]. In addition, Ordovician bioerosion trace fossils include bivalve borings (*Petroxestes*), bryozoan etchings (*Ropalonaria*), sponge borings (*Cicatricula*), *Sanctum* (a cavernous domichnium excavated in bryozoan zoaria by an unknown borer) and *Gastrochaenolites* [8], [9]. Bioerosion was very common in the Middle Paleozoic, especially in the Devonian [10]. Later in the Mesozoic bioerosion intensity and diversity further increased [6], [9], [11], [12], and deep, large borings became especially common [13].

The bioerosion trace fossils of Ordovician of North America are relatively well studied [14], [15], [16]. In contrast, there is a limited number of works devoted to the study of bioerosional trace fossils in the Ordovician of Baltica. The earliest large boring occurs in the Early to Middle Ordovician hardgrounds and could belong to *Gastrochaenolites* [4], [17]. Abundant *Trypanites* borings are known from brachiopods of the Arenigian [18] and Sandbian [19]. Wyse Jackson and Key [20] published a study on borings in trepostome bryozoans from the Ordovician of Estonia. They identified two ichnogenera, *Trypanites* and *Sanctum,* in bryozoans of Middle and Upper Ordovician strata of northern Estonia.

The aims of this paper are to: 1) determine whether the shafts in large Sandbian bryozoans belong to previously known or a new bioerosional ichnotaxon for the Ordovician; 2) determine the systematic affinity of the trace fossil; 3) discuss the ecology of the trace makers; 4) discuss the paleobiogeographic distribution of the trace fossil; and 4) discuss the occurrence of large borings during the Ordovician Bioerosion Revolution.

### Geological Background and Locality

During the Ordovician, the Baltica paleocontinent migrated from the temperate to the subtropical realm [21], [22]. The climatic change resulted in an increase of carbonate production and sedimentation rate on the shelf during the Middle and Late Ordovician. In the Upper Ordovician the first carbonate buildups are recorded, emphasizing a striking change in the overall character of the paleobasin [23].

The total thickness of the Ordovician in Estonia varies from 70 to 180 m [23]. The Ordovician limestones of Estonia form a wide belt from the Narva River in the northeast to Hiiumaa Island in the northwest [23]. In the Middle Ordovician and early Late Ordovician, the slowly subsiding western part of the East-European Platform was covered by a shallow, epicontinental sea with little bathymetric differentiation and an extremely low sedimentation rate. Along the extent of the ramp a series of grey calcareous - argillaceous sediments accumulated (argillaceous limestones and marls), with a trend of increasing clay and decreasing bioclasts in the offshore direction [22].

**Figure 1:**
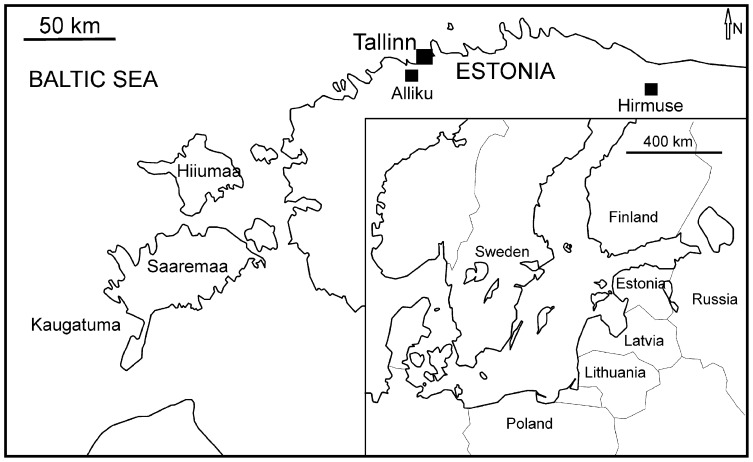
Locality map. Location of Hirmuse Creek and Alliku Ditches in North Estonia.

**Figure 2:**
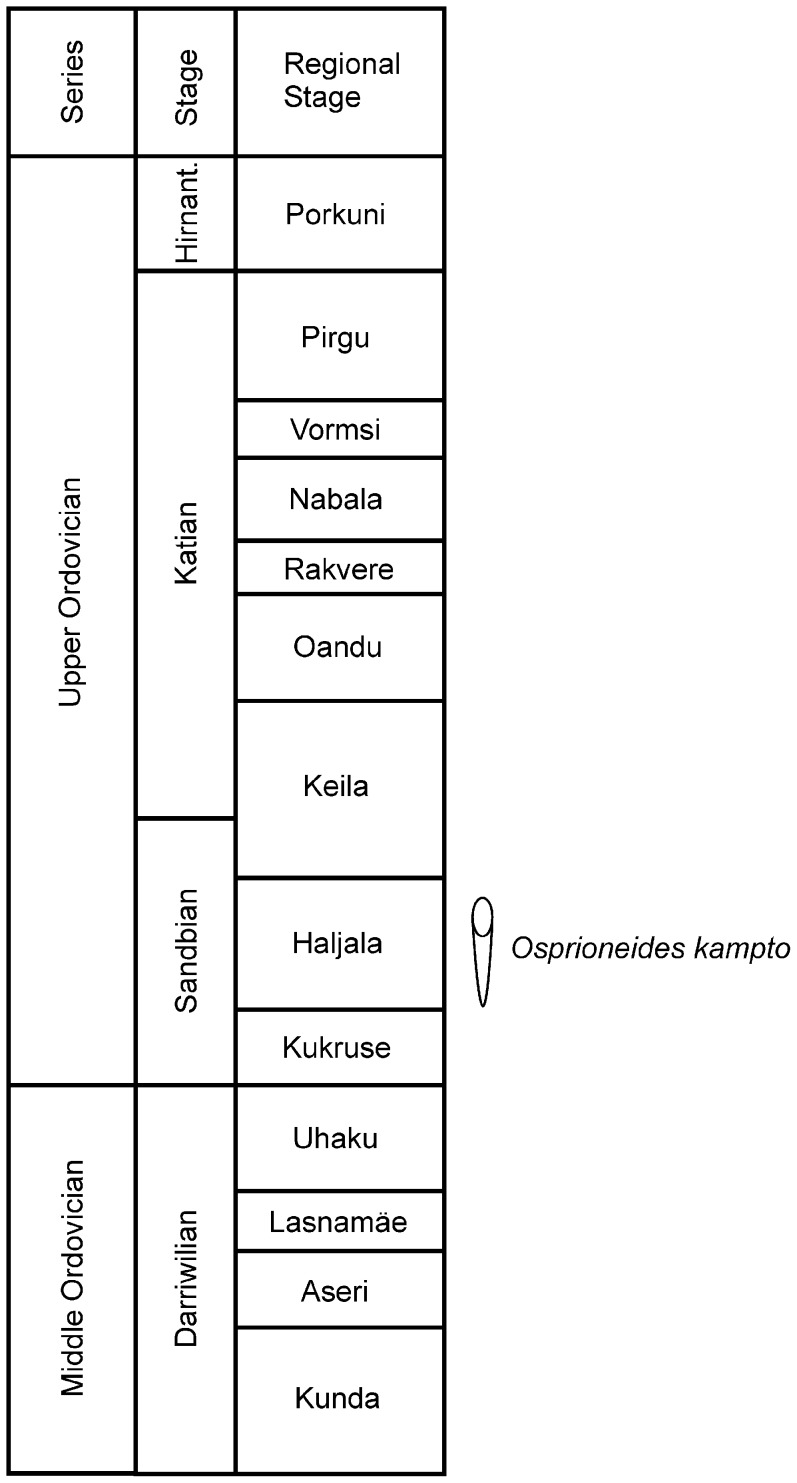
Stratigraphic scheme. The Middle and Upper Ordovician in Estonia. Location of *Osprioneides kampto* borings. Modified after Hints et al. (2008).

The material studied here was collected from the Hirmuse Creek (Fig. 1) and Alliku Ditches (Fig. 1) of Sandbian age (Haljala Stage) (Fig. 2). Hirmuse Creek is located in Maidla parish of Ida-Viru County. Clayey and skeletal limestones with interlayers of marls are exposed on the creek bed and in its banks. The fossil assemblage includes algae (*Mastopora*), brachiopods (*Clinambon*, *Leptaena*, *Platystrophia*, *Apartorthis*, *Porambonites*, *Pseudolingula*), conulariids, gastropods (*Lesueurilla*, *Holopea*, *Bucanella*, *Pachydictya*, *Megalomphala*, *Cymbularia*), ichnofossils (*Amphorichnus*, *Arachnostega*), sponges, receptaculitids (*Tettragonis*), rugosans (*Lambelasma*), bryozoans, and asaphid trilobites. The Alliku Ditches are located in Harju County near the village of Alliku. Clayey limestones with interlayers of marls are exposed here. The fauna includes algae, brachiopods, bryozoans (*Aluverina*, *Annunziopora*, *Batostoma*, *Ceramoporella*, *Coeloclema*, *Constellaria*, *Corynotrypa*, *Crepipora*, *Dekayella*, *Diazipora*, *Diplotrypa*, *Enallopora*, *Esthoniophora*, *Graptodictya*, *Hallopora*, *Hemiphragma*, *Homotrypa*, *Homotrypella*, *Kukersella*, *Lioclemella*, *Mesotrypa*, *Monotrypa*, *Nematopora*, *Nematotrypa*, *Oanduella*, *Moyerella*, *Pachydictya*, *Phylloporina*, *Prasopora*, *Proavella*, *Pseudohornera*, *Rhinidictya*, *Stictoporella*), echinoderms, gastropods, ostracods, rugosans, receptaculitids and trilobites according to Rõõmusoks [24].

No permits were required for the described study, which complied with all relevant Estonian regulations, as our study did not involve collecting protected fossil species. Three described bryozoan specimens with the *Osprioneides* borings are deposited at the Institute of Geology, Tallinn University of Technology (GIT), Ehitajate tee 5, Tallinn, Estonia, with specimen numbers GIT-398-729, GIT 665-18 and GIT 665-19.

## Results

**Figure 3:**
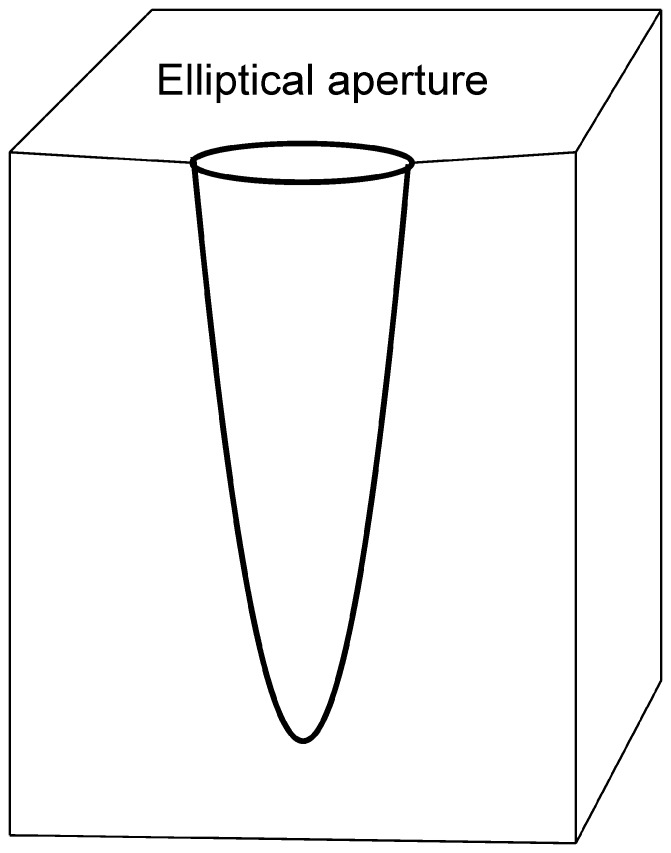
*Osprioneides kampto.* Schematic line drawing showing a straight boring.

**Figure 4:**
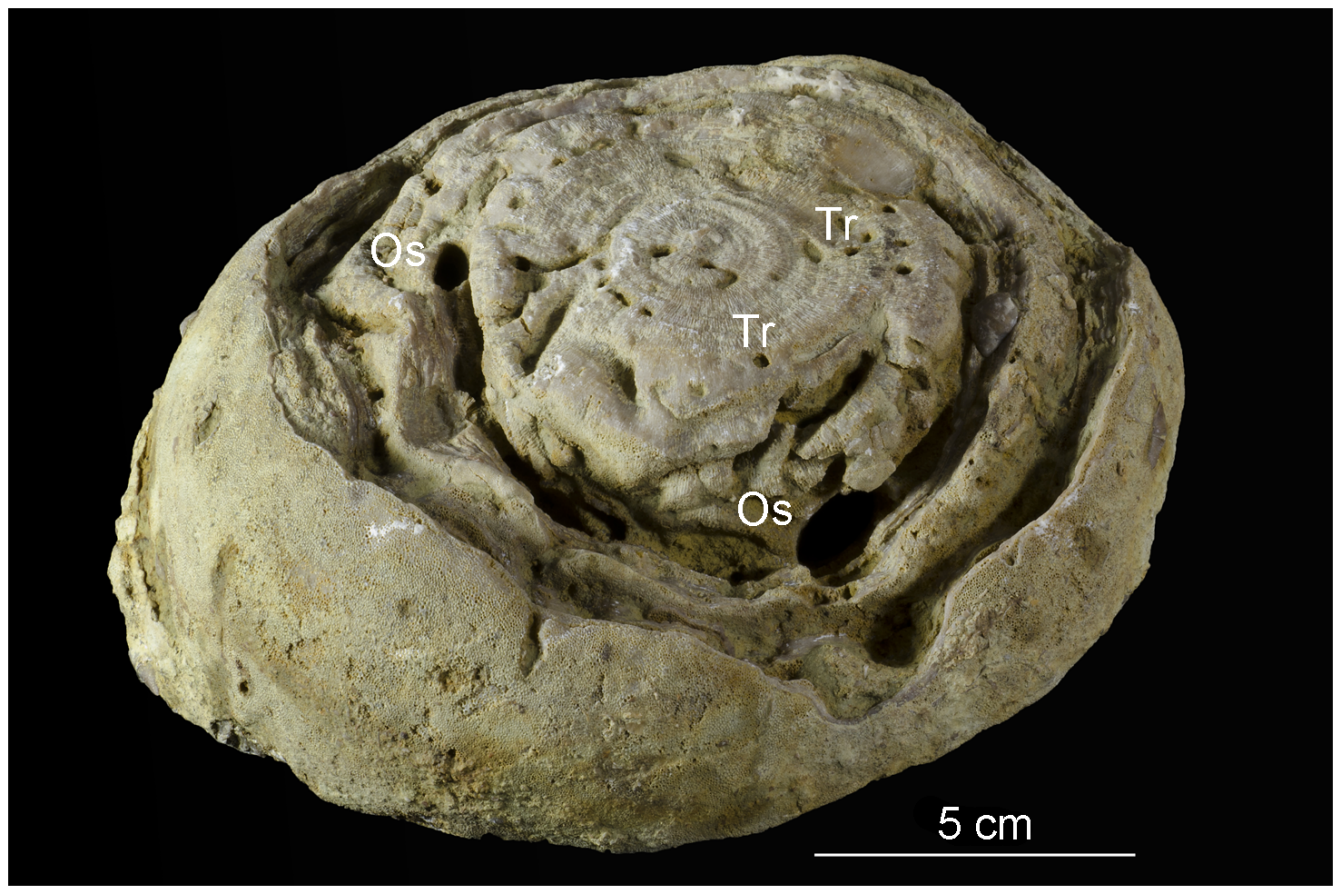
*Osprioneides kampto* borings (Os). A bryozoan from Hirmuse Creek, Sandbian, Upper Ordovician, Estonia. Tr – *Trypanites* borings. GIT 398–729.

**Figure 5:**
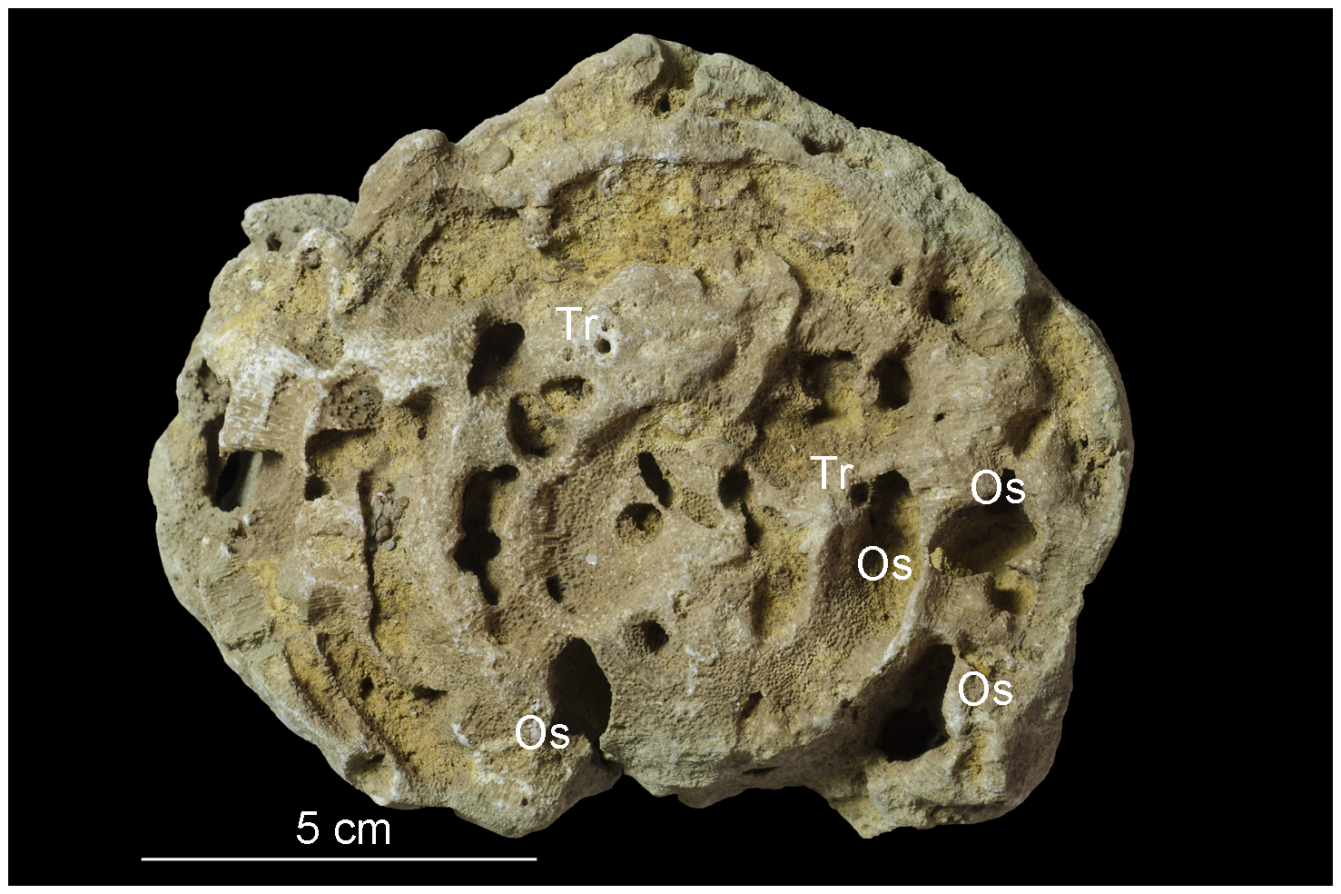
*Osprioneides kampto* borings (Os). A bryozoan from Hirmuse Creek, Sandbian, Upper Ordovician, Estonia. Tr – *Trypanites* borings. GIT 665-18.

**Figure 6:**
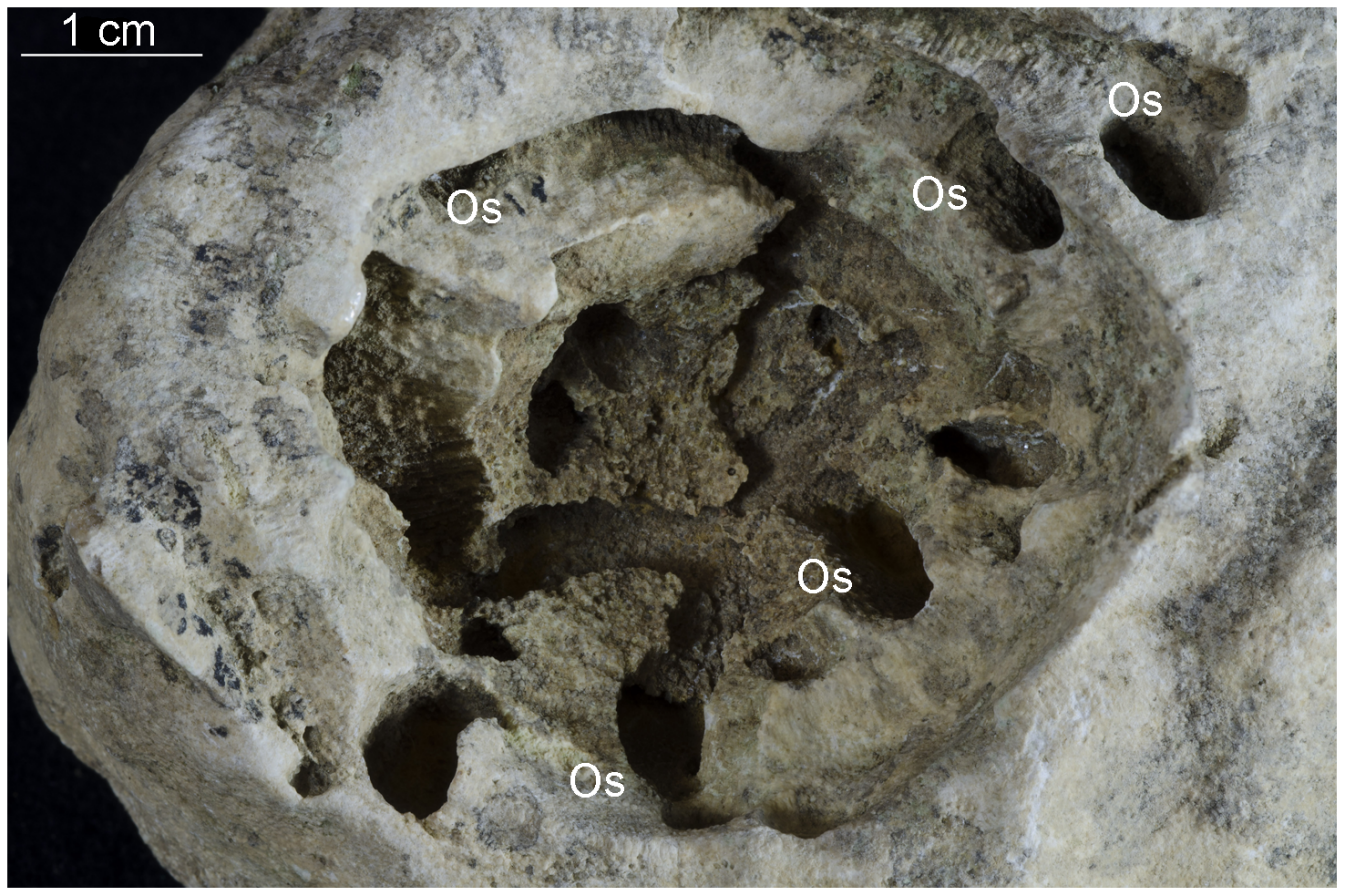
*Osprioneides kampto* borings (Os). A bryozoan from Hirmuse Creek, Sandbian, Upper Ordovician, Estonia. Tr – *Trypanites* borings. GIT 665-19.

Numerous unbranched, single-entrance, large deep borings with oval cross sections were found in three large trepostome bryozoan colonies (Figs. 3, 4, 5, 6). The borings are vertical to subparallel to the bryozoan surfaces and have a tapered to rounded terminus. Several borings have lost their roofs due to erosion. The boring apertures’ minor axis is 2.7 to 7.0 mm (M = 5.05, sd = 1.34, N = 12) and major axis is 7.0 to 15.0 mm (M = 10.37, sd = 2.60, N = 12) long. The axial ratio (major axis/minor axis) of the borings ranges from 1.60 to 2.59 (M = 2.08, sd = 0.29, N = 12). Three completely preserved borings are 25 mm (aperture 12×6 mm), 28 mm (aperture 9×4.5 mm), and 32 mm (aperture 13×6 mm) deep. Two unroofed borings have depths of 35 mm and 50 mm. The borings are abundant in the studied samples (Figs. 3, 4, 5, 6). They occasionally truncate each other, which somewhat resembles a branching pattern. There are no linings or septa inside the borings. The growth lamellae of the bryozoans show no reactions around the borings. Small *Trypanites* borings occur inside the large boring with oval cross section. The apertures of the large borings occur on both the upper and lower surfaces of the bryozoans (the upper and lower surface of bryozoans was determined by looking at skeletal growth).

## Discussion

### Taxonomic Affinity of the Borings and the Possible Trace Maker

The borings in these bryozoans resemble somewhat *Petroxestes* known from Late Ordovician bryozoans and hardgrounds of North America [14]. Both are of unusually large size for Ordovician borings, and both have oval-shaped apertures. However, in *Petroxestes* the aperture width is much greater than the boring’s depth. In contrast, the depth of the borings in bryozoans is much greater than their apertural width. Unlike *Petroxestes*, the Sandbian borings examined here have a tapering terminus and somewhat sinuous course. The axial ratio of *Petroxestes* borings aperture (major axis/minor axis) is also much greater than observed in these borings.

The other similar large Palaeozoic boring is *Osprioneides*, which is known from the Silurian of Baltica, Britain and North America [13]. We assign borings in the bryozoans studied here to *Osprioneides kampto* because of their similar general morphology. They have a single entrance, an oval cross section, and significant depth similar to *Osprioneides kampto*. Their straight, curved to somewhat sinuous shape also resembles that of *Osprioneides*. Both *Osprioneides* and these borings in bryozoans have a tapered to rounded terminus.

Most likely the *Osprioneides* trace maker was a soft-bodied animal similar to polychaete worms that used chemical means of boring as suggested by Beuck et al. [13]. This is supported by the slightly curved to sinuous course of several borings and their variable length. The presence of a tapered terminus in *Osprioneides* means bivalves were very unlikely to have been the trace makers.

### Paleoecology and Taphonomy

*Osprioneides* borings were made post mortem because the growth lamellae of the bryozoan do not deflect around the borings. There are also no signs of skeletal repair by the bryozoans. Several *Osprioneides* borings truncate other *Osprioneides* borings that were likely abandoned by the trace maker by that time. Similarly, empty *Osprioneides* borings were colonized by *Trypanites* trace makers. This indicates that the *Osprioneides* borings may have appeared relatively early in the ecological succession. Overturning of the bryozoan zoaria can explain the occurrence of *Osprioneides* borings apertures on both upper and lower surfaces. There is no sign of encrustation on the walls of the studied *Osprioneides* borings, suggesting relatively rapid burial of the host bryozoans shortly after the *Osprioneides* colonization.

It is likely that *Osprioneides* trace makers were suspension feeders similar to the *Trypanites* animals due to their stationary life mode [25]. Bryozoan skeletons may have offered them protection against predators and a higher tier for suspension feeding. Previously known host substrates of *Osprioneides* comprise stromatoporoids and tabulate corals. This new occurrence of *Osprioneides* borings in large bryozoans shows that the trace maker possibly selected its substrate only by size of skeleton because the traces are not found in smaller fossils. However, they are not found in any Ordovician hardgrounds that provide more area than do the bryozoan colonies. Wyse Jackson and Key [20] suggest that large bryozoan colonies were exploited by borers because they would have been easy to bore into.

### Ordovician Bioerosion Revolution

Morphological diversification was not the only result of the Ordovician Bioerosion Revolution. Most of the large bioerosional traces of the Paleozoic had their earliest appearances in the Ordovician [7], [8]. The earliest known large borings are those of *Gastrochaenolites* from the Early Ordovician of Baltica [4], [17]. Later, during the Late Ordovician, large *Petroxestes* borings appeared in North America. At the same time the *Osprioneides* borings described here appeared in Baltica. Thus the Ordovician was also the time of great increase in quantities of hard substrate removed by single trace makers. The biological affinities of Ordovician *Gastrochaenolites* are not known [8], but it may have been a soft-bodied animal. The Late Ordovician *Petroxestes* was almost certainly produced by the facultatively boring bivalve *Corallidomus scobina* [26]. Boring polychaetes were the likely *Osprioneides* trace makers, which is suggested by the somewhat sinuous shape of some borings. This indicates that more than one group of animal was involved in the appearance of large bioerosional traces during the Ordovician Bioerosion Revolution. Increased predation pressure [27] was most likely the driving force behind the infaunalization of larger invertebrates such as the *Osprioneides* trace makers in the Ordovician. On the other hand in echinoids, for example, infaunalization was presumably the result of colonization of unoccupied niche space [28].

### Paleobiogeography

*Osprioneides* is a relatively rare fossil compared to the abundance of *Trypanites* in the Silurian of Baltica [29]. In the Silurian, *Osprioneides* borings also occur outside of Baltica. They are known from the Llandovery of North America and Ludlow of the Welsh Borderlands [30]. *Osprioneides* is presumably absent in the Ordovician of North America because Ordovician bioerosional trace fossils of North America are relatively well studied [15], [16]. Thus, it is possible that the *Osprioneides* trace maker originated in Baltica or elsewhere and migrated to North America in the Silurian. This may well be connected to the decreased distance between Baltica and Laurentia (the closing of the Iapetus Ocean) and the loss of provinciality of faunas in the Silurian.
